# Immune Modulating Capability of Two Exopolysaccharide-Producing *Bifidobacterium* Strains in a Wistar Rat Model

**DOI:** 10.1155/2014/106290

**Published:** 2014-05-29

**Authors:** Nuria Salazar, Patricia López, Pablo Garrido, Javier Moran, Estefanía Cabello, Miguel Gueimonde, Ana Suárez, Celestino González, Clara G. de los Reyes-Gavilán, Patricia Ruas-Madiedo

**Affiliations:** ^1^Department of Microbiology and Biochemistry of Dairy Products, Instituto de Productos Lácteos de Asturias-Consejo Superior de Investigaciones Científicas (IPLA-CSIC), Paseo Río Linares s/n, Villaviciosa, 33300 Asturias, Spain; ^2^Department of Functional Biology, Immunology Area, University of Oviedo, C/Julián Clavería s/n, Oviedo, 33006 Asturias, Spain; ^3^Department of Functional Biology, Physiology Area, University of Oviedo, C/Julián Clavería s/n, Oviedo, 33006 Asturias, Spain

## Abstract

Fermented dairy products are the usual carriers for the delivery of probiotics to humans, *Bifidobacterium* and *Lactobacillus* being the most frequently used bacteria. In this work, the strains *Bifidobacterium animalis* subsp. *lactis* IPLA R1 and *Bifidobacterium longum* IPLA E44 were tested for their capability to modulate immune response and the insulin-dependent glucose homeostasis using male Wistar rats fed with a standard diet. Three intervention groups were fed daily for 24 days with 10% skimmed milk, or with 10^9^ cfu of the corresponding strain suspended in the same vehicle. A significant increase of the suppressor-regulatory TGF-**β** cytokine occurred with both strains in comparison with a control (no intervention) group of rats; the highest levels were reached in rats fed IPLA R1. This strain presented an immune protective profile, as it was able to reduce the production of the proinflammatory IL-6. Moreover, phosphorylated Akt kinase decreased in gastroctemius muscle of rats fed the strain IPLA R1, without affecting the glucose, insulin, and HOMA index in blood, or levels of Glut-4 located in the membrane of muscle and adipose tissue cells. Therefore, the strain *B. animalis* subsp. *lactis* IPLA R1 is a probiotic candidate to be tested in mild grade inflammation animal models.

## 1. Introduction

Probiotics, together with the prebiotic substrates that support the growth of the beneficial intestinal microbiota, constitute one of the largest segments of the worldwide functional food market. Fermented foods, and especially dairy products, are the most popular carriers for the delivery of these microorganisms in humans [1]. Probiotics are defined as “live microorganisms that, when administered in adequate amounts, confer a health benefit on the host” [2]. Strains from* Bifidobacterium* and* Lactobacillus* are frequently used as probiotics for humans; some of their species have the “Qualified Presumption of Safety” (QPS) status [3] because of their long history of safe consumption.

There are several reports supporting the fact that certain ingested probiotics are able to impact the human health by direct interaction with the host cells, or through the modulation of the intestinal microbiota [4, 5]. The relevance of this microbiota community is especially highlighted in some chronic disorders of the gut in which a dysbiosis of this microbial community has been detected [6]. In addition, scientific evidence suggests an intricate relationship between the intestinal microbiota and some extraintestinal disorders, such as obesity. The modulation of the gut microbiota by diet could be effective in improving the low-grade inflammation associated with obesity and related diseases [7, 8]. Prebiotic and probiotic supplements could modify the altered gut microbiota present in obesity-associated diseases by influencing gut barrier function, insulin sensitivity, systemic inflammation, and host energy homeostasis [9, 10]. The mechanism(s) by which probiotics interact with the host remains to be completely understood, although some clues have been obtained from studies performed using different animal models [11–13].

Surface components of probiotic envelopes are claimed to be the molecules that establish the initial interaction with eukaryotic cells. In this scenario, exopolysaccharides (EPS) produced by members of the intestinal microbiota, or by beneficial microorganisms ingested with foods, can be active players. There are a few works studying* in vivo* the involvement of these polymers on bacteria-host interactions [14–16]. Most of the evidence of the immune modulation capability of EPS from probiotics has been obtained by* in vitro* approaches. It seems that the physicochemical characteristics, such as composition (mainly the presence of charged substituents) and molecular weight (size), of these polymers are the key parameters determining the capability to induce a mild response (acid and small polymers) or to reduce the production of cytokines (neutral and big polymers) [17]. In parallel to the direct interaction with immune cells of the host, the immunomodulation could also be achieved through intervention on the intestinal microbiota [18, 19]. Previously we have demonstrated that the administration of the EPS-producing strains* Bifidobacterium animalis* IPLA-R1 and* Bifidobacterium longum* IPLA-E44 to male Wistar rats modified their intestinal microbiota by influencing the short chain fatty acid (SCFA) profile and by increasing* Bifidobacterium* population levels in the gut [15]. Therefore, the aim of the current study was to check whether the oral intake of these two EPS-producing bifidobacteria could modify some health-related parameters, such as the systemic inflammatory profile and/or the insulin-dependent glucose homeostasis, in healthy rats fed with a standard diet. The final goal is to suggest target human population(s) for the potential application of these strains as probiotics.

## 2. Material and Methods

### 2.1. Experimental Design and Samples Collection

The animal study design was previously reported [15] and was conducted under the approval of the Animal Experimentation Ethical Committee of Oviedo University (Asturias, Spain). The EPS-producing strains* B. animalis* subsp.* lactis* IPLA-R1 and* B. longum* IPLA-E44 were tested in adult, male Wistar rats. Briefly, three groups of rats (8 per group) were fed daily, through an intragastric cannula, with the delivery vehicle (100 *μ*L skimmed milk, group V) or with 10^9^ cfu per day (in 100 *μ*L skimmed milk) of the strains IPLA-R1 (group B1) or IPLA-E44 (group B2). After an intervention period of 24 days, animals were anaesthetized with halotone and killed by exsanguination. Additionally, a group of 8 rats was used as a basal reference control (no intervention, group C) and killed under the same conditions.

Blood samples (4 mL) were collected from the jugular vein into heparinized tubes and centrifuged at 1,000 ×g for 20 min at 4°C, and the plasma fraction was immediately collected and stored frozen at −20°C until it was assayed. The gastrocnemius muscle and retroperitoneal adipose tissue (100 mg) were dissected, frozen in liquid nitrogen, and kept at −80°C until the analyses.

### 2.2. Immunoglobulins and Cytokine Profile in Plasma

The cytokine levels in the plasma samples were quantified by a “cytometric bead array” (CBA) using the BD FascCanto II flow cytometer and the software FCAP (BD Biosciences, San Diego, CA, USA). The CBA flex set (BD Biossciences) included the cytokines IL-1a, IL-4, IL-6, IL-10, IFN*γ*, and TNF*α*, which were assayed under conditions recommended by the manufacturer. The TGF*β* was measured by means of the eBioscience platinum ELISA test (eBioscience, Bender MedSystems GmbH, Vienna, Austria); the colorimetric reaction was measured at 450 nm in the modulus microplate photometer (Turner Biosystems, CA, USA). The limit of detection was 4.0 pg/mL for IL-1a, 3.4 pg/mL for IL-4, 1.6 pg/mL for IL-6, 19.4 pg/mL for IL-10, 6.8 pg/mL for IFN*γ*, 27.7 pg/mL for TNF*α*, and 8 pg/mL for TGF*β*.

The levels of immunoglobulin (Ig) IgG and IgA were determined by means of ELISA tests (GenWay Biotech, Inc., San Diego, CA, USA) following the manufacturer's instructions. Additionally, IgA was measured in supernatants obtained after centrifugation from fecal samples homogenized (1/10) with PBS.

### 2.3. Determination of Insulin, Glucose, and Calculation of the HOMA-Index

The tail vein blood glucose levels were measured using a portable device (Accu-Chek Aviva Nano System, Roche Farma, S.A., Barcelona, Spain) while fasting plasma insulin was measured by ELISA assay (Millipore Ibérica, S.A., Madrid, Spain) following the manufacturer's recommendations. Homeostasis Assessment Model- (HOMA–) index was calculated using the following formula: [insulin (*μ*U/mL) × glucose (mg/dL)]/2.43 [20].

### 2.4. Analysis of the Protein Kinase B (Akt) and the Glucose Transporter Type 4 (Glut4)

The content of total and phosphorylated Ser473 Akt kinase, as well as that of the insulin-regulated glucose transporter type 4 (Glut4), was determined by means of western-blot analyses in samples of crude intracellular extracts and in cell-membrane fractions, obtained from the muscle and retroperitoneal adipose tissues of the rats as follows. To obtain the intracellular crude extracts, both tissue types were homogenized in lysis buffer (50 mM Tris-HCl pH 7.5, 150 mM NaCl, 1% Nonidet P40, 0.05% sodium deoxycholate, sodium orthovanadate, 5 mM EDTA, and 10% glycerol) at 4°C. The homogenized samples were centrifuged at 21,800 ×g at 4°C for 10 min to collect the supernatants (crude extracts) and its protein content was determined by the Bradford method. To obtain cell membrane fractions, a modification of the method described by Hirshman et al. [21] was used. Briefly, a total of 500 mg of tissues was homogenized with a Polytron operated at maximum speed for 30 s at 4°C in a buffer containing 100 mM Tris (pH 7.5), 20 mM EDTA (pH 8.0), and 255 mM sucrose (pH 7.6). The homogenate was then centrifuged at 1,000 ×g for 5 min and the resulting supernatant was centrifuged again at 48,000 ×g for 20 min. The pellet from this centrifugation was used for the preparation of the membrane fraction that is enriched in the membrane marker Na^+^-K^+^-ATPase. The pellet was resuspended in 20 mM HEPES and 250 mM sucrose, pH 7.4 (buffer A). An equal volume of a solution containing 600 mM KCl and 50 mM sodium pyrophosphate was added and the mixture was vortexed, incubated for 30 min on ice, and then centrifuged for 1 h at 227,000 ×g over a 36% sucrose cushion in buffer A. The resulting interface and the entire buffer above it were collected, diluted in an equal amount of buffer A, and centrifuged for 1 h at 227,000 ×g. The resulting pellet was used as the cell membrane fraction and its protein content was determined by the Bradford method.

To carry out the western-blot analysis, proteins in the crude tissue extracts or in the cell membrane fractions were resolved by SDS-PAGE (10% Tris-Acrylamide-Bis) and electrotransferred from the gel to nitrocellulose membranes (Hybond-ECL, Amersham Pharmacia, Piscataway, NJ) as described by Towbin et al. [22]. Nonspecific protein binding to the nitrocellulose membrane was reduced by preincubating the filter with blocking buffer (TNT, 7% BSA); then, membranes were incubated overnight with the primary antibodies Glut4 (sc-7938, diluted 1 : 2,500), Akt (sc-7126, diluted 1 : 2,000), and phosphorylated-Ser473-Akt (sc-101629, diluted 1 : 2,500). All antibodies were obtained from Santa Cruz Biotechnologies (Santa Cruz, CA). After incubation with the primary antibody, the nitrocellulose membranes were washed and incubated with the corresponding anti-rabbit antibody coupled to horseradish peroxidase (HRP, sc-2004, diluted 1 : 20,000), or the anti-goat antibody coupled to HRP (sc-2768, diluted 1 : 20,000). Additionally, all membranes were stripped and probed with monoclonal antibodies used as reference controls: anti-*β*-actin antibody (sc-1615, diluted 1 : 2,500), anti-Na^+^-K^+^-ATPase *α*1-subunit antibody (sc-16041, diluted 1 : 5,000), or anti-GAPDH (sc-20356, diluted 1 : 1,000). Immunoreactive bands were detected using an enhanced chemiluminescence system (ECL, Amersham Pharmacia Biotech, Little Chalfont, Bucks, UK). Films were analyzed using a digital scanner Nikon AX-110 (Nikon, Madrid, Spain) and NIH Image 1.57 software (Scion Corp., MD, USA). The density of each band was normalized to its respective loading control (*β*-actin, ATPase, or GAPDH). In order to minimize interassay variations in each experiment, samples from all animal groups were processed in parallel.

### 2.5. Statistical Analysis

The SPSS/PC 19.0 software package (SPSS Inc., Chicago, IL, USA) was used for all statistical analyses. After checking the normal distribution of the parameters involved in the homeostasis of glucose, one-way ANOVA tests were used to determine the differences between the three groups of rats and the reference control. Moreover, differences among the three experimental groups, compared two by two, were also tested by means of one-way ANOVA tests. These parameters were represented by mean and standard deviation (SD).

Data of cytokines and Igs were not normally distributed; thus, the nonparametric Mann-Whitney test for two independent samples was used to assess differences. The same comparisons among samples previously described were carried out. Cytokine data were represented by median, interquartile range and maximum and minimum values (box and whiskers plot).

## 3. Results

### 3.1. Immune Parameters

**Figure 1:**
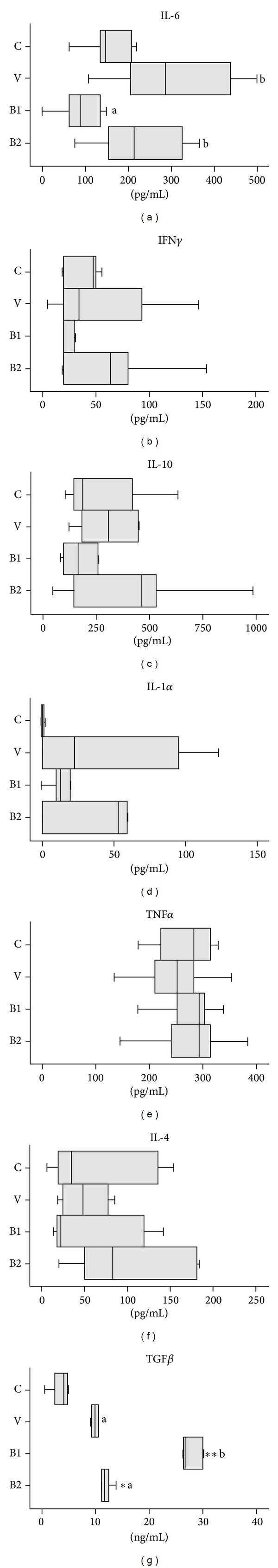
Cytokines measured in blood (plasma) samples of Wistar rats fed for 24 days with vehicle (100 *μ*L of skimmed milk, V group) or 10^9^ cfu per day of* B. animalis* subps.* lactis* IPLA-R1 (B1 group) or* B. longum* IPLA-E44 (B2 group). The control rats were not submitted to the intervention study (C group). For each cytokine, the box and whiskers plot represents median, interquartile range and minimum and maximum values obtained from 8 rats per group. The nonparametric Mann-Whitney test for two independent samples was used to compare each treatment group with the control, and differences are indicated with asterisks (*P < 0.05, **P < 0.01). Additionally, the same test was used to assess differences among the treatment groups compared two by two. In this case, treatment groups that do not share the same letter are statistically different (*P* < 0.05).

Several proinflammatory and immune-suppressor cytokines were measured in the blood plasma obtained from the four groups of rats (Figure 1). Levels of most cytokines (IFN*γ*, IL-1*α*, IL4, IL-10, and TNF*α*) remained without significant variations in the four groups of rats; this indicates that the daily intake for 24 days of the two bifidobacteria, or the vehicle (milk), has not strongly modified the immune response, since most of the cytokine levels in the intervention groups (V, B1, and B2) were similar to those found in the control group (C). In spite of this, the oral intake of the two bifidobacteria significantly increased the production of the suppressor-regulatory TGF-*β* cytokine, the levels reached with the strain* B. animalis* subsp.* lactis* IPLA-R1 (group B1) being the highest (*P* < 0.05). In addition, this strain also induced the lowest (*P* < 0.05) production of IL-6 as compared with the other two intervention (V and B2) groups, although none of the three intervention groups significantly differed from the control group. Thus, it seems that the strain IPLA-R1 showed an* in vivo* immune suppressive profile by reducing the proinflammatory cytokine IL-6 and inducing the synthesis of the regulatory TGF-*β*.

**Figure 2:**
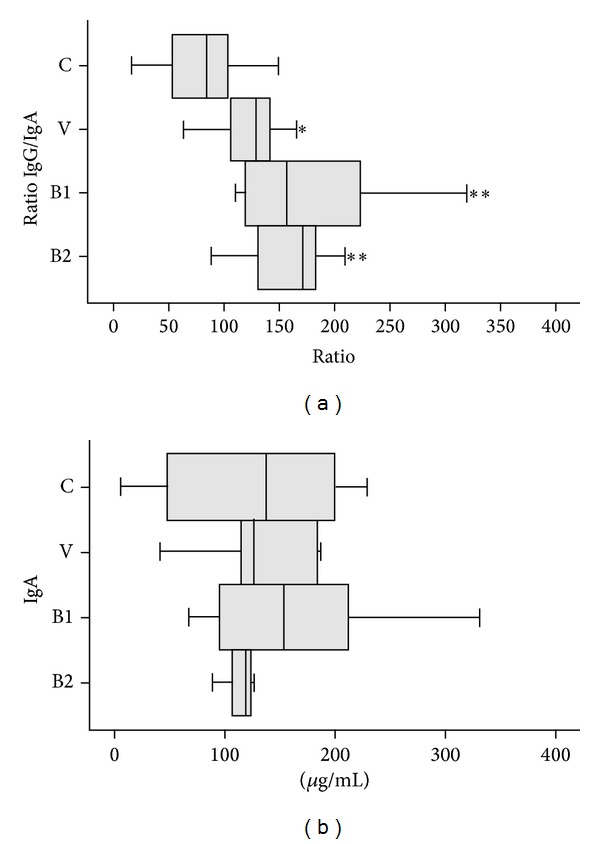
Ratio IgG/IgA in blood (plasma) samples (a) and amount of IgA (*μ*g/mL) secreted in fecal samples (b) of Wistar rats fed for 24 days with vehicle (100 *μ*L of skimmed milk, V group) or 10^9^ cfu per day of* B. animalis* subps.* lactis* IPLA-R1 (B1 group) or* B. longum* IPLA-E44 (B2 group). The control rats were not submitted to the intervention study (0 days). The same statistical treatment indicated in Figure 2 was applied.

The levels of IgA were determined in blood plasma and fecal homogenates and the amount of IgG was measured in plasma. The oral intake of skimmed milk, alone or used as vehicle for the bifidobacterial delivery, produced a significantly higher (*P* < 0.05) ratio IgG/IgA in the three groups, in comparison with the basal control group (Figure 2(a)). No variations in secretory IgA were detected in the fecal samples of the four groups of rats (Figure 2(b)), which is of special relevance since this antibody plays a critical role in maintaining the immune homeostasis in several mucosae, including the intestinal mucosa. Therefore, (cow's) milk induced a humoral systemic response; this immune reaction was not surprising since this food is not a current component of a rat's diet, and therefore these animals have not developed oral tolerance to it.

### 3.2. Biochemical Parameters

The current setup of data showed that the concentration of glucose and insulin in plasma collected after a fasting period, as well as the HOMA index, were not modified by the intervention study (Table 1). The concentrations in the groups of rats treated for 24 days with vehicle (skimmed milk), or with the two bifidobacteria, were similar among them and with respect to the control group.

**Figure 3:**
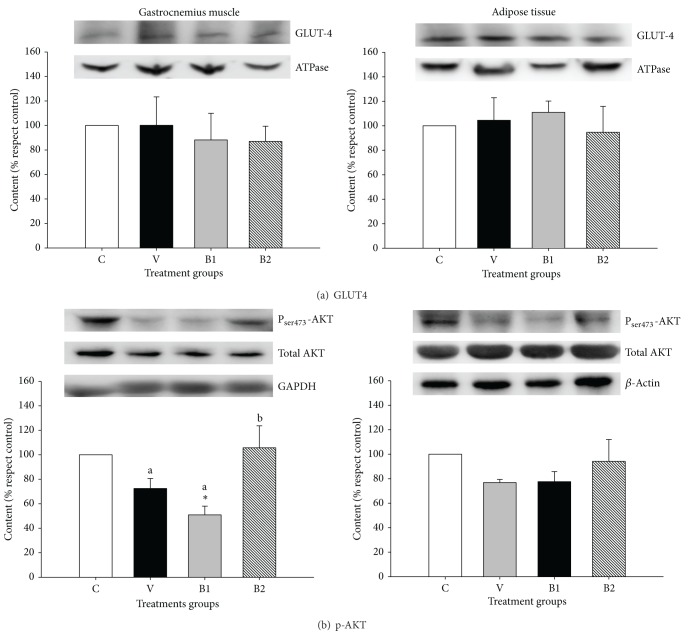
Content of the cell-membrane Glut4 (a) as well as the intracellular Akt and phosphorylated-Ser_473_-Akt (b) in gastrocnemius muscle and adipose tissues from rats fed daily for 24 days with delivery vehicle (100 *μ*L of skimmed milk, V group) or 10^9^ cfu per day of* B. animalis* subps.* lactis* IPLA-R1 (B1 group) or* B. longum* IPLA-E44 (B2 group). Data were referred to those obtained in the control rats (C group) which were not submitted to the intervention study. Bars represent mean and standard deviations obtained from 8 rats per group. Independent one-way ANOVA tests were used to compare each treatment group with the control, and differences are indicated with asterisks (*P < 0.05). Additionally, the same test was used to assess differences among the treatment groups compared two by two. In this case, treatment groups that do not share the same letter are statistically different (*P* < 0.05).

To detect potential changes in the insulin-dependent glucose signaling route, the levels of the protein Akt and the glucose transporter Glut4 were quantified by western blot (Figure 3). The levels of glucose transporter Glut4 located in the cellular membrane of both retroperitoneal adipose tissue and gastrocnemius muscle were similar in all groups of rats (Figure 3(a)). Similarly, no statistical differences were detected in the percentage of the intracellular kinase Akt, phosphorylated in the serine 473 residue, in adipose tissue (Figure 3(b)). However, the phosphorylated-Akt was significantly (*P* < 0.05) lower in the gastrocnemius muscle of rats fed for 24 days with* B. animalis* subsp.* lactis* IPLA-R1 (group B1) in comparison with the other two intervention groups (vehicle or* B. longum* IPLA E44 fed), as well as in comparison with the control group.

## 4. Discussion

In recent years, there is an increasing evidence that some specific probiotic strains are able to modulate the immune response. In the case of* Bifidobacterium* genus, most strains studied showed an anti-inflammatory profile in animal models genetically modified or challenged with different factors to induce an inflammatory process [23–25]. Our experimental model was performed with standard, naïve (not challenged) Wistar rats that simulate a healthy state. Thus, this could be the main reason why most cytokines tested were not significantly modified by the ingestion of the two bifidobacteria, in comparison with the placebo fed rats. However, it should also be taken into account that both bifidobacteria are producers of EPS; these are polymers that could mask other immune-reactive molecules present in the bacterial surface and therefore allow them to escape the immune system survey. In this regard, Fanning and coworkers [14] have demonstrated in a naïve murine model that the EPS-producing* Bifidobacterium breve* UCC2003 strain failed to elicit a strong immune response in comparison to its EPS-deficient variant strains; it seems that the EPS+ strain is able to evade the B-cell response. We have recently demonstrated that bifidobacterial EPS, differing in their physicochemical composition,* in vitro* induced a variable cytokine production pattern by human peripheral blood mononuclear cells [26]. In general, those EPS having high molecular weight were those eliciting the lowest production of any cytokine [27, 28]. Thus, it seems that not only the presence/absence of the polymer, but also the characteristics intrinsic to each EPS are relevant for their capability to induce immune response. In this regard the two bifidobacteria strains used in the current work produce polymers of different chemical composition [15]; only the group of rats receiving the strain* B. animalis* subsp.* lactis* IPLA R1 showed a significantly reduced production of IL-6 and increased synthesis of TGF-*β*. The differential immune response elicited by the two strains cannot be exclusively attributed to the production of different EPS, since other strain-associated traits could also be responsible. Nevertheless, it seems that IPLA R1 strain is able to elicit an imunosuppressive profile* in vivo* after oral intake for a prolonged period (24 days).

Regarding the glucose homeostasis, the levels of circulating glucose and insulin, as well as the HOMA index, were not modified by the consumption of the two bifidobacteria in the context of a standard (no high fat, no high carbohydrate) diet. In this regard, it has been described that some probiotics can improve the resistance to insulin in different animal models of diet-induced diabetes or with different genetic backgrounds [29–32]. Additionally, a double-blind, randomized intervention study in humans showed that an intake of* Lactobacillus acidophilus* NCFM for 4 weeks improved the insulin sensitivity [33]. In most of these reports no mechanism of action is proposed or is a general one suggested, such as the modulation of the intestinal microbiota, or the modification of the inflammatory state. In our study, we checked some critical points in the cascade of the glucose uptake mediated by insulin, such as the location of the glucose transporter Glut4 and the levels of the active (phosphorylated) Akt kinase [34]. The two EPS-producing bifidobacteria strains tested did not modify the insulin-regulated trafficking of the glucose transporter Glut4 from intracellular vesicles (endosomes) to the cell membrane of either adipose or muscular tissues. The failure of this translocation in response to insulin is one of the steps in the development of insulin resistance and type 2 diabetes. Therefore, the presence of similar Glut4 levels in the cell membrane of tissues obtained from the four groups of rats explains the absence of variations in the levels of circulating glucose and insulin. One of the proteins involved in the insulin-mediated Glut4 trafficking is the phosphatidylinositol 32-kinase (PI 3K)-dependent Ser473 kinase Akt. In response to insulin, Akt is activated by phosphorylation which directs the traffic of Glut4 from vesicles to the cell membrane; therefore, Akt acts as a regulator of glucose transport [35]. In our experimental model, the intracellular levels of phosphorylated-Akt in adipocytes were not significantly modified by the intake of the two bifidobacteria; this result is consistent with the absence of differences in the amount of Glut4 located in the cell membrane, as well as the lack of variation in circulating glucose, among the four groups of rats. However, the percentage of phosphorylated-Akt was significantly lower in the gastrocnemius muscle of rats fed with the strain* B. animalis* subsp.* lactis* IPLA R1. Since, in rats from this group, the glucose homeostasis parameters and the content of the Glut4 located in the cell membrane of muscle and adipose tissue remained without significant variations, differences in the phosphorylated-Akt could be explained by the participation of this kinase in other metabolic routes apart from the insulin-mediated glucose transport. In this regard, it has been indicated that the PI 3K-dependent Ser/Thr kinase Akt is a regulator that acts in many different metabolic routes and several events related with the cellular cycle [35].

Aiming to have a general picture of the differences detected in our experimental model, which were mainly driven by the strain* B. animalis* subsp.* lactis* IPLA R1, it should be pointed out that levels of circulating IL-6 and phosphorylated-Akt in muscle were directly related. In this regard, the skeleton muscle and the adipose tissue are important sources for systemic IL-6 [36]. In addition, during strong exercise muscular cells are also targets for the action of IL-6, where the insulin action is favored, among other events, by enhancing the phosphorylation of Akt [37]. However, IL-6 has adverse effects on other tissues that are targets for insulin action, such as the liver and adipose tissue [38]. At present, we cannot establish a hypothesis to explain the relationship between systemic IL-6 and phosphorylated-Akt in muscle found in rats fed* B. animalis* subsp.* lactis* IPLA R1. Nevertheless, recent articles show that Akt activity has a role in regulating immune response since it is involved in the differentiation and response of several cellular subsets, such as T cells and macrophages [39, 40]. The activity of Akt in signaling immune pathways is induced in some cases by the presence of bacterial components, such as the lipopolysaccharide from gram-negatives [41] or peptidoglycan from gram-positives [42]. This kinase also plays a role in the innate immunity signaling, since it participates in the modulation of mucin secretion by intestinal epithelial cells in response to pathogens [43]. Furthermore, the activity of Akt has been associated with dendritic cell differentiation and stimulation driven by Gram-positive probiotics, such as the strain* Bifidobacterium breve* C50 [44].

## 5. Conclusion

In this study, we found that the oral administration of the EPS-producing* B. animalis* subsp.* lactis* IPLA R1 in healthy rats is associated with an immune protective profile, since this EPS producing strain can suppress the proinflammatory cytokine IL-6 and promote the synthesis of the regulatory cytokine TGF-*β*. These results suggest that, in the future, this bifidobacteria could be tested in experimental models of low grade inflammation state, such as that linked to obesity. Additionally, the capability of strain IPLA R1 to reduce the systemic levels of IL-6, linked with a reduction in the phosphorylated state of Akt in the muscle, without affecting the glucose homeostasis, prompts us to propose the potential application of this strain for sportspeople undertaking strong exercise.

## Figures and Tables

**Table 1:** Parameters related to the glucose homeostasis measured in the plasma of Wistar rats fed for 24 days with vehicle (100 *μ*L of skimmed milk) or 10^9^ cfu per day of  *B. animalis* subps. *lactis* IPLA-R1 (B1 group) or *B. longum* IPLA-E44 (B2 group). Control rats were not submitted to the intervention study (0 days). The one-way ANOVA analyses did not show statistical differences.

Rat group	Mean ± SD		
	Glucose (mg/dL)	Insulin (*μ*g/mL)	HOMA
Control (0 d)	76.2 ± 15.4	0.0060 ± 0.0045	0.20 ± 0.091
Vehicle (24 d)	74.3 ± 12.3	0.0061 ± 0.0052	0.21 ± 0.093
B1 (24 d)	69.6 ± 12.3	0.0063 ± 0.0049	0.19 ± 0.089
B2 (24 d)	82.4 ± 7.9	0.0063 ± 0.0051	0.17 ± 0.090
